# Hepatitis B Vaccination among 1999–2017 Birth Cohorts in Zhejiang Province: The Determinants Associated with Infant Coverage

**DOI:** 10.3390/ijerph15122915

**Published:** 2018-12-19

**Authors:** Yu Hu, Yaping Chen, Ying Wang, Hui Liang

**Affiliations:** Institute of Immunization and Prevention, Zhejiang Provincial Center for Disease Control and Prevention, Hangzhou 310000, China; ypchen@cdc.zj.cn (Y.C.); ywang@cdc.zj.cn (Y.W.); hliang@cdc.zj.cn (H.L.)

**Keywords:** hepatitis B vaccine, vaccination, birth cohort, vaccination coverage, determinant

## Abstract

This study aimed to investigate the coverage of hepatitis B vaccine (Hep B) and its completeness and timeliness for birth cohorts from 1999 to 2017 in Zhejiang province, East China. Demographic characteristics and vaccination records of Hep B of children born from 1 January 1999 to 31 June 2017 were extracted from the Zhejiang provincial immunization information system. The timeliness of the first dose of Hep B (Hep B1) was defined as the proportion of children who received the Hep B1 within 24 h after birth among the target population. The completeness of Hep B was defined as the proportion of children who completed the three-dose series of Hep B before 7 years of age. The demographic characteristics of the target population were described. The coverage of each dose of Hep B, the timeliness of Hep B1, and the completeness of Hep B for each birth cohort were described. A logistic regression model was applied to detect the determinants of the timeliness of Hep B1 and the completeness of Hep B vaccination. The coverage of Hep B1 increased from 90.3% to 98.3%, the coverage of Hep B2 increased from 88.8% to 96.1%, and the coverage of Hep B3 increased from 86.4% to 94.2%. The timeliness of Hep B1 increased from 80.3% to 91.3%. The completeness of Hep B increased from 81.3% to 91.5%. The determinants of timeliness of Hep B1 included children’s delivery place, immigration status, maternal education level, and economic development level of resident area. The determinants of completeness of Hep B included children’s delivery place, immigration status, maternal education level, economic development level of resident area, maternal occupation, and frequency of vaccination service. Zhejiang province had achieved significant improvements in the timely administration of Hep B1 and the completeness of Hep B. To accelerate progress toward additional reductions in the transmission of hepatitis B virus, further efforts need to be focused on improving the timeliness of Hep B1 vaccination and reducing the drop-outs among disadvantaged children with the risk factors identified in this study.

## 1. Introduction

Hepatitis B is a viral infection that spreads through the blood or bodily fluids. Globally, approximately 2 billion people are currently infected by hepatitis B virus (HBV), and an estimated 240 million people are chronic HBV carriers. More than 780,000 people die every year due to the consequences of HBV infection, such as hepatic failure, cirrhosis, and hepatocellular carcinoma [1]. The probability of developing a chronic infection is inversely related to the age of infection. Almost 80–90% of infants infected during the first year of life develop the chronic infection [1]. As such, the prevention of mother-to-child transmission has been crucial to reducing HBV’s prevalence. Vaccination of hepatitis B vaccine (Hep B), which has been demonstrated to be safe and effective, is considered as the key intervention to reduce the disease burden associated with HBV infection. The World Health Organization (WHO) [1] recommends that the first dose of Hep B (Hep B1) be given as early as possible after birth, ideally within the first 24 h.

HBV infection is a serious health problem in China, causing a substantial disease burden. The national serum epidemiological survey of HBV in 1992 indicated that the prevalence of hepatitis B surface antigen (HBsAg) was 9.8% in the general population, and 9–12% in children under 5 years of age [2]. Since perinatal transmission is a major mode of HBV transmission in China, the Chinese government made strong efforts to establish universal childhood Hep B vaccination in line with the recommendations of WHO [3]. The recommended schedule involves the administration of Hep B1 within 24 h after birth and the administration of two subsequent doses at 1 and 6 months of age. This scheme can not only effectively prevent mother-to-child transmission, but also provide lifelong protection [4].

The Chinese policy on Hep B vaccination has undergone some changes in the last two decades. In 1992, the Chinese Ministry of Health (MoH) recommended that children’s Hep B be classified as a category two vaccine, which required the parents to cover the expense. User fees charged to parents by local health departments for the vaccine’s purchase and its administration were two serious barriers to uptake at that time. Hep B has been included in the Chinese expanded program on immunization (CEPI) since 2002 and provided to all children for free, while parents were still required to pay the administration fee. Hep B1 should be administrated in the first 24 h at the delivery hospital, according to the “who delivers the infant gives the birth dose” policy. It meant that the hospital who delivered the infant had the responsibility to ensure that Hep B1 was given in a timely manner. Meanwhile, the Chinese MoH recommended the screening of all pregnant women for HBsAg, and demanded that all infants born to HBsAg-positive mothers should have administered the Hep B1 and hepatitis B immunoglobulin simultaneously within 24 h of birth. Since 2007, all vaccinations for children under 15 years of age, including Hep B, have been offered without any charge, and the amount of HBsAg of Hep B increased from 5 ug to 10 ug. Since 2016, the latest Chinese vaccination schedule updated the recommendations of Hep B for low-birth-weight infants and premature infants. It stipulated that Hep B1 should be administered to all newborns even if they were low-weight or premature. Hep B1 should not be counted in the routine schedule among infants with a low birth weight or premature infants, and these infants should restart the three-dose schedule at one month of age. Alongside the improvements in Hep B vaccination policy, extensive efforts on maternal health and other aspects of the public health service were made in the same period, including an increase in the hospital delivery rate and staff training, the development of a local vaccination strategy, and improvements on the awareness of HBV infection. As a result, both the coverage for Hep B vaccination and the timeliness of Hep B1 increased dramatically from 1992 to 2012 [5]. Meanwhile, the carrier rate in Chinese children under 5 years of age fell to 0.96% in 2006, and further to 0.32% in 2014 (from 10% in 1992) [6]. The reduction of 90% met the goals set by WHO [1].

Zhejiang province is located in East China, with 70 million inhabitants living in 11 cities and an annual birth cohort of approximately 700,000 newborns. Just like the whole nation, the carrier rate of HBsAg in Zhejiang has decreased remarkably in the general population and in children under 5 years of age since the introduction of Hep B into CEPI in 2002. However, previous studies had always evaluated the coverage of the individual Hep B dose at a cross-sectional level and did not focus on the completeness and the timely administration of Hep B1 [2,7,8]. Furthermore, the trends of coverage before and after the introduction of Hep B into CEPI as well as the influence of policy changes have not been completely described. Therefore, this study aimed to investigate the vaccination coverage, completeness, and timeliness of Hep B for the birth cohorts from 1999 to 2017. We also intended to add to the existing knowledge on the development of the childhood Hep B vaccination strategy in China.

## 2. Methods

### 2.1. Data Resources

We obtained data from the Zhejiang provincial immunization information system (ZJIIS), which consists of two parts: client software and central server. ZJIIS is deployed in the Zhejiang provincial health network, which is a local area network that is isolated from the Internet to protect the data’s privacy while covering the center for disease control and prevention (CDC) at different administrative levels and immunization clinics. Any data extracted from ZJIIS should be de-identified.

The client software is deployed in immunization clinics and delivery rooms in the maternity hospitals. The client software is used to collect demographic information (such as name, gender, date of birth, delivery place, family address, phone number, immigration status, maternal occupation, and maternal education level), and vaccination records. All of this information will be uploaded to the central server in real time or updated if there is any change, and can be downloaded or shared among different vaccination clinics in Zhejiang Province. It is worth noting that the client software deployed in delivery rooms can create a record of every newborn after his/her birth, which includes the demographic information of the child and his/her mother and the vaccination record of Hep B1. When a child is brought to the vaccination clinic at one month old to receive the next vaccinations, the staff of immunization clinics can download his/her vaccination record from the center server before making new records on it. For migrant children from other provinces, their demographic information and the complete historic vaccination records are required to be entered into the client software when they first visit any immunization clinic in Zhejiang province. Additionally, the information on the frequency of sessions and working time of immunization clinics are also uploaded to the central server. The frequency of sessions means the number of times an immunization clinic has been open during a specific time period (e.g., once per month, two times per week).

The central server is deployed in Zhejiang Provincial CDC. It receives, stores, and processes all of the data collected from the client software, and makes them exchangeable among different immunization clinics. To date, over 10 million records of children born from 1 January 1999 are stored in the central server of ZJIIS. The “Zhejiang Provincial Immunization Information Management Platform (ZJIISMP)” is a website that can help staff of CDCs at different administrative levels to monitor the process and outcome of the EPI work. ZJIISMP can generate plenty of tables and figures, including vaccination coverage rates by vaccine or by dose, completeness and timeliness of vaccination for a specific birth cohort, drop-out rates, and lists of unvaccinated children.

### 2.2. Target Population

Children born from 1 January 1999 to 31 June 2017 and registered in ZJIIS were enrolled in this study. Appropriately anonymized individual records of children, including Hep B vaccination records, were extracted from ZJIIS on 1 July 2018. The 12-month-wide cohort method was adopted to calculate the vaccination coverage of each dose of Hep B, the timeliness of Hep B1, and the completeness of the three-dose series of Hep B. For example, the 1999 cohort consisted of children born between 1 January 1999 and 31 December 1999. However, the 2017 cohort consisted of children born between 1 January 2017 and 31 June 2017. The reason for only including half the birth cohort of 2017 in our analyses was that we needed to ensure that every child had the opportunity to finish the three-dose series of Hep B.

### 2.3. Study Areas

Zhejiang province consists of 11 cities in total. Since Huzhou city did not participate in ZJIIS, our analyses were performed in the remaining 10 cities. In 2016, the number of newborns of Zhejiang province was 739,650, and the number of newborns of Huzhou was 23,311, with a proportion of 3.2%. The 10 cities were classified into three strata according to the Gross Domestic Product (GDP) per capita, by using the census data of 2017. Hangzhou (HZ), Ningbo (NB), and Zhoushan (ZS) were classified as the high level for GDP per capita >16,000 USD; Shaoxing (SX), Jinhua (JH), and Jiaxing (JX) were classified as the middle level for GDP per capita between 10,000 and 16,000 USD; and Taizhou (TZ), Quzhou (QZ), Lishui (LS), and Wenzhou (WZ) were classified as the low level for GDP per capita <10,000 USD.

### 2.4. Definitions

The coverage was defined as the proportion of vaccinated individuals among the target population. The timeliness of Hep B1 was defined as the proportion of individuals who received Hep B1 within 24 h after birth among the target population. The completeness of Hep B was defined as the proportion of individuals who completed the three-dose series of Hep B before 7 years of age. The second dose of Hep B (Hep B2) was considered to be invalid if it was administrated <28 days from Hep B1. The third dose of Hep B (Hep B3) was considered to be invalid if it was administrated <60 days from Hep B2 [9]. Invalid doses were considered to be non-vaccinations.

### 2.5. Statistical Analysis

All analyses were performed with STATA MP version 14.0 (StataCorp. 2015, Stata statistical software, college station, TX, USA). The demographic characteristics were described by different birth cohorts. The coverage of each dose of Hep B, the timeliness of Hep B1, and the completeness of Hep B were estimated by city and birth cohort, along with the 95% confidence interval (CI). In order to detect the determinants of the timeliness of Hep B1 and the completeness of Hep B vaccination, the generalized mixed linear model was adopted to account for the clustered nature of the data from ZJIIS (nested within cities) and the binary response of the outcome variable. The city was treated as a random effect, and other demographic characteristics were treated as fixed in our analyses. The Final model was: $\mathrm{logit}\left(P_{ij}\right)=\beta_{0}+\beta 1x_{ij}+\beta 2x_{ij}+\ldots +\beta nx_{ij}+\mu_{j}+\epsilon_{ij}$, where P*_ij_*: probability of the i^th^ child in the j^th^ community to be immunized in a timely manner with Hep B1 or be complete with the Hep B series, *β_0_*: intercept, *β_n_*: regression coefficient, x*_ij_*: independent variables, u*_j_*: community level errors, and e*_ij_*: individual-level errors. All variables on demographics, the economic status of resident areas, and vaccination service provision were included in the final models. The adjusted odds ratio (AOR) with 95% CI for each variable was calculated. The statistical significance was set at 0.05.

### 2.6. Ethical Considerations

This study was exempt from ethical review since it involved the assessment of de-identified data.

## 3. Results

In total, 10,911,530 children were enrolled in this analysis for the birth cohorts dating from 1999 to 2017. The male-to-female ratio was 1.03. Of the enrolled children, 92.9% were delivered in hospital, and 42.3% were migrant. The proportion of mothers with fixed jobs and with an education background of senior middle school or above was 87.1% and 90.4%, respectively. Almost 40% of the enrolled children were from areas with the high economic development level, and almost 16% of the enrolled children received the vaccination service from clinics with a service frequency of 5 times per week or more (Table 1).

Table 2 presents the vaccination coverage of Hep B, the timeliness of Hep B1, and the completeness of Hep B for the birth cohorts from 1999 to 2017. The coverage of Hep B1 increased from 90.3% (the 1999 birth cohort) to 98.3% (the 2016 birth cohort), the coverage of Hep B2 increased from 88.8% (the birth cohort 2000) to 96.1% (the birth cohort 2014), and the coverage of Hep B3 increased from 86.4% (the birth cohort 2000) to 94.2% (the birth cohort 2016). The timeliness of Hep B1 increased from 80.3% (the birth cohort 2001) to 91.3% (the birth cohort 2017). The completeness of Hep B increased from 81.3% (the birth cohort 2000) to 91.5% (the birth cohort 2017).

The results from the two generalized mixed linear models are presented in Table 3. Determinants of the timeliness of Hep B1 included children’s delivery place, immigration status, maternal education level, and economic development level of resident area. All coefficients that were statistically significant in the timeliness of Hep B1 were also significant in the completeness of Hep B and had the same sign. Additionally, maternal occupation and frequency of vaccination service were also significantly associated with the completeness of Hep B. The variance at city level in two models remained significant (*p* < 0.01) after controlling for other factors. The values of intra-class correlation (ICC) were 17.2% for the model of timeliness of Hep B1 and 15.7% for the model of completeness of Hep B, showing the relevant proportions of the variation due to the clustered nature of the data.

## 4. Discussion

There are numerous studies on the development of the Chinese Hep B vaccination program [2]. These studies mainly focused on the vaccination strategies among infants or children, which was reasonable because of the elevated risk of severe consequences of HBV infections for the relevant age groups. Our study added to the literature by offering an independent estimate from the immunization information system at the provincial level.

Generally, the coverage of each dose of Hep B improved from the birth cohort 1999 to the birth cohort 2017 and had maintained the goal of 90% since the birth cohort 2004. This trend was similar to that at the national level [2,7,10]. The coverage of the three-dose Hep B tripled from 30.0% for children born in 1992 to 91% for children born in 2012 at the national level [2]. As a result of this improvement, the carrier rate in children under 5 years of age fell to 0.32% in 2014 (from 10% in the 1990s) and the reduction rate of 90% met the goal set by WHO [1]. We inferred that there were some reasons for the improvement of Hep B vaccination. First, in 2002, the central government removed the vaccine cost, added Hep B into the routine vaccination schedule, and gave it to all children for free. The vaccination cost is considered to be one of the most important barriers to immunization worldwide. In Zhejiang province, we had found that the coverage of the varicella vaccine, which was introduce in the late 1990s, reached a plateau of coverage of up to 74.8% in 2014 and was significantly lower than the coverage of any vaccine included in CEPI [11]. Second, the awareness of Hep B vaccination was enhanced for both parents and providers through health education or training [12,13]. Third, the policy requiring ‘who deliver the infant, who has to vaccinate the Hep B1 within 24 h’ might be attributed to the increase of Hep B vaccination coverage [2,10]. Additionally, the Chinese government has, since 2009, provided incentives to pregnant women to give birth in hospital, which would also improve the hospital delivery rate [3]. Although there had been a great improvement in infant Hep B coverage, this study showed that certain groups remained at high risk of not receiving a timely birth dose or full immunization. This was consistent with previous reports from other provinces of China [4,14,15,16,17], and was attributed to risk factors such as uneven economic development, rural areas, and ethnic minorities. Other reports from some developing countries attributed the delayed or incomplete Hep B vaccination to the poor practice of the vaccination service [18,19,20]. For example, Smith et al. found that some health workers were initially reluctant to immunize children with false contraindications, such as a low birth weight, a low-grade fever, or other mild diseases [21].

In this study, we found the timeliness of Hep B1 had increased from 81.2% to 91.3%. It was in line with the trend of this indicator at the national level, which increased from 22.2% for the 1992 birth cohort to 90% for the 2012 birth cohort [2]. In the two generalized mixed linear models, similar associations with the timeliness of Hep B1 and the completeness of Hep B were found. Previous studies from China had reported a high male-to-female ratio in some rural areas, which resulted from a combination of sex-selective abortion and mistreatment of girls [22,23,24]. Due to the neglect of girls, a lower coverage or a more delayed vaccination could be expected among girls than among boys. However, our findings suggested that the timeliness of Hep B1 and the completeness of Hep B did not vary significantly between sexes. Hospital delivery is considered to be a positive factor for both the timeliness and completeness of vaccinations worldwide [25,26]. As we know, the administration of Hep B1 at birth has been required for every registered maternity hospital since 2002, and the rate of hospital delivery has increased rapidly in the last two decades [27]. Hence, assuming a mother delivers at a maternity hospital, her child will receive Hep B1 in a timely manner. Furthermore, those mothers who deliver their infants in hospital are closer to the public health service and may have a better utilization of the vaccination service [13], which will improve the completeness of Hep B. Migrants were more likely to have an incomplete or a delayed Hep B vaccination, which did not replicate the patterns of the utilization of the vaccination service among resident children [28]. The coverage of oral polio vaccine (OPV) was 85.3% and the timeliness of the first dose of OPV was 39.7% along the Thailand–Myanmar Border [29]. These estimates were significantly lower than the targets set by WHO. Here, we inferred that the missed opportunities or delayed vaccinations of Hep B1 might be associated with the vulnerability of migrants in adjusting to a new socio-cultural environment, the service demand, and the satisfaction with the previous experience [30,31,32]. However, the exact reasons need further exploration due to the limitations of the study design.

An occupied mother was an inverse factor of the completeness of Hep B vaccination. It was consistent with our previous study, and we assumed that they did not have enough time to spare for primary healthcare and were less aware of the information on vaccination [13,33]. A higher maternal education background was positively associated with both the timeliness of Hep B1 and the completeness of Hep B, which is in line with the previous reports [13,34]. A higher education background would help a mother communicate with the health workers smoothly and have a positive influence on the vaccination practice through a better understanding of and accepting the knowledge or policy [35]. Suarez-Castaneda et al. had found that children whose caregivers had achieved secondary education were more likely to receive a timely vaccination (AOR = 1.53) than those whose caregivers had no formal education [36]. Children from undeveloped areas were most likely to have a delayed or an incomplete vaccination, which is consistent with a previous report, showing that poverty-related factors hinder the utilization of vaccination services [32,37]. This is probably due to the poor accessibility caused by indirect costs (such as the transport cost or the cost of medication for non-serious adverse events) or a deduction from wages for work leave for a child’s vaccination. Our study showed that, even in a province that could be considered to be uniformly developed, a small difference in economic development could impact on the utilization of a vaccination service. The frequency of sessions also played an important role in the completeness of Hep B, which was consistent with the previous studies [7,38]. It was demonstrated that frequent sessions and extending the service time were the most beneficial interventions for working mothers through improving the convenience and accessibility of the service and enhancing mothers’ compliance with the vaccination schedule [39,40,41,42].

Our analyses supplemented the previous studies based on the nationwide serum surveys, the administrative coverage aggregated at the county level, or a combination of different data sources. This approach also had a wider scope than household surveys from limited geographical regions. This study has some limitations. First, our findings were based on 10 cities, and could not be generalized to the entire province. Second, our analyses included the children registered in ZJIIS, and the children who were not registered might have a lower coverage for all vaccines [33]. Based on a previous study conducted in Zhejiang province [43], the registration rate of ZJIIS among children aged 24–35 months was 87.8%. This means that 12.6% of the eligible children were not covered by ZJIIS. As a result, we would have overestimated the coverage. Third, not all risk factors mentioned in previous reports for Hep B vaccination could be explored due to the limitations in the data from ZJIIS.

## 5. Conclusions

Zhejiang province has made a great achievement in integrating Hep B into routine immunization programs, achieving significant improvements in the timely administration of Hep B1 and the completeness of Hep B. However, there were still some children with a delayed Hep B1 or an incomplete Hep B vaccination. To reduce the prevalence of HBV infection, and to accelerate progress toward additional reductions in HBV transmission, further efforts need to be focused on improving the timely administration of Hep B1 and reducing the number of drop-outs among disadvantaged children with the risk factors identified in this study.

## Figures and Tables

**Table 1:** The demographic characteristics of birth cohorts from 1999 to 2017, Zhejiang province.

Variables		Birth Cohort																		
		1999	2000	2001	2002	2003	2004	2005	2006	2007	2008	2009	2010	2011	2012	2013	2014	2015	2016	2017 *
No. of children		512,078	519,202	512,148	500,566	517,325	518,672	538,233	554,477	582,751	596,949	593,180	604,630	610,375	647,803	642,309	701,688	681,230	716,339	361,575
Gender (%)	Male	51.1	51.2	51.4	50.8	50.9	50.4	49.8	50.6	50.6	49.9	50.7	50.6	50.7	51.1	50.7	50.6	50.5	50.4	49.8
	Female	48.9	48.8	48.6	49.2	49.1	49.6	50.2	49.4	49.4	50.1	49.3	49.4	49.3	48.9	49.3	49.4	49.5	49.6	50.2
Delivery place (%)	Hospital	85.4	86.7	87.9	88.4	90.1	91.8	92.6	92.4	93	93.5	94.2	94.8	95.1	95.4	95.3	95.2	95.8	96.4	96.1
	Home	14.6	13.3	12.1	11.6	9.9	8.2	7.4	7.6	7	6.5	5.8	5.2	4.9	4.6	4.7	4.8	4.2	3.6	3.9
Children’s immigration status (%)	Migrant	20.9	22.9	26.8	30.8	33.9	38.4	39.8	41.8	45.5	50.6	51.5	49.6	50.6	52.8	50.6	47.3	46.2	45.2	44.8
	Resident	79.1	77.1	73.2	69.2	66.1	61.6	60.2	58.2	54.5	49.4	48.5	50.4	49.4	47.2	49.4	52.7	53.8	54.8	55.2
Maternal occupation	Home fulltime	10.8	12.8	11.8	10.6	11.5	10.8	11.8	11.5	13.8	12.8	10.9	12.8	13.5	15.4	15.8	14.9	14.7	13.8	13.2
	Employed	89.2	87.2	88.2	89.4	88.5	89.2	88.2	88.5	86.2	87.2	89.1	87.2	86.5	84.6	84.2	85.1	85.3	86.2	86.8
Maternal education level	<senior middle school	17.5	15.5	14.5	14.3	13.8	13.4	12.7	11.2	10.8	9.2	8.4	7.5	7.2	6.4	5.9	4.7	4.9	5.1	4.6
	≥senior middle school	82.5	84.5	85.5	85.7	86.2	86.6	87.3	88.8	89.2	90.8	91.6	92.5	92.8	93.6	94.1	95.3	95.1	94.9	95.4
Socioeconomic level of resident areas ^#^ (%)	Low	26.7	27.1	27.4	27.5	27.5	27.8	27.6	27.4	27.1	27.6	27.3	27.3	27.5	28.0	27.1	27.6	27.4	27.2	27.5
	Middle	32.5	32.0	33.0	33.0	32.5	32.7	32.2	32.7	33.0	32.9	32.4	32.6	32.8	32.8	32.7	32.9	32.4	32.2	33.0
	High	40.8	40.9	39.6	39.5	40.0	39.5	40.2	40.0	39.9	39.5	40.3	40.2	39.8	39.2	40.2	39.5	40.2	40.6	39.5
Frequency of vaccination service (%)	≤3 times per month	72.6	71.5	70.6	70.5	68.5	66.7	64.5	60.8	54.4	52.9	51.2	46.2	40.5	37.5	32.5	26.4	22.9	18.1	17.6
	1–4 times per week	24.3	25.7	25.9	25.9	25.7	28.6	26	27.7	32.2	32.3	33.1	33	37.7	40.1	42.9	46.8	49.9	52.4	50.6
	≥5 times per week	3.1	2.8	3.5	3.6	5.8	4.7	9.5	11.5	13.4	14.8	15.7	20.8	21.8	22.4	24.6	26.8	27.2	29.5	31.8

*: 2017 cohort were children born between 1 January and 31 June 2017. ^#^: Hangzhou (HZ), Ningbo (NB), and Zhoushan (ZS) were classified as high economic development areas; Shaoxing (SX), Jinhua (JH), and Jiaxing (JX) were classified as middle economic development areas; and Taizhou (TZ), Quzhou (QZ), Lishui (LS), and Wenzhou (WZ) were classified as low economic development areas.

**Table 2:** The vaccination coverage, the timeliness of the first dose of hepatitis B vaccine (Hep B1), and the completeness of hepatitis B vaccination (Hep B) for birth cohorts from 1999 to 2017, Zhejiang province.

Birth Cohort	Coverage (%)			Timeliness of Hep B1 (%)	Completeness (%)
	Hep B1	Hep B2	Hep B3		
1999	90.3	89.4	87.2	81.2	82.4
2000	91.5	88.8	86.4	80.9	81.3
2001	90.7	89.2	87.6	80.3	82.4
2002	91.8	90.4	89.4	82.7	83.8
2003	92.3	90.7	89.8	84.2	84.6
2004	93.3	91.4	90.2	85.7	86.3
2005	94.2	91.8	90.2	86.3	87.3
2006	94.8	92.8	91.1	86.6	89.4
2007	95.1	92.4	91.2	86.9	89.9
2008	95.7	93.6	92.4	87.2	89.7
2009	96.5	94.0	92.5	88.5	90.2
2010	96.9	94.6	93.0	89.4	90.6
2011	97.2	95.7	93.8	90.1	91.1
2012	97.3	95.3	94.1	90.6	91.4
2013	97.4	95.4	93.2	91.2	90.9
2014	98.2	96.1	93.6	91.3	91.1
2015	97.6	95.2	94.1	90.9	91.4
2016	98.3	95.4	94.2	90.9	90.8
2017 *	97.9	95.3	93.6	91.3	91.5
Total	95.3	93.2	91.6	87.4	88.5

*: The 2017 cohort was children born between 1 January and 31 June 2017.

**Table 3:** Determinants associated with timeliness of Hep B1 and the completeness of Hep B for birth cohorts from 1999 to 2017, Zhejiang province.

Variables	No. of Children	Timeliness of Hep B1		Completeness of Hep B	
		%	AOR (95% CI)	%	AOR (95% CI)
**Fixed effects**					
Gender (%)					
Male	5,524,117	87.4	1	88.5	1
Female	5,387,413	87.5	1.1 (0.9–1.0)	88.5	1.0 (1.0–1.1)
Delivery place (%)					
Hospital	10,134,444	94.0	1	94.9	1
Home	777,086	2.0	0.4 (0.3–0.7) **	4.7	0.5 (0.3–0.8) **
Children’s immigration status (%)					
Migrant	4,618,771	81.0	1	82.6	1
Resident	6,292,759	92.2	2.4 (1.9–3.6) *	92.8	2.4 (1.6–3.7) *
Maternal occupation					
Home fulltime	1,411,842	87.6	1	93.2	1
Employed	9,499,688	87.4	1.0 (0.9–1.0)	87.8	0.7 (0.6–0.9) *
Maternal education level					
< senior middle school	1,043,141	68.0	1	71.4	1
≥ senior middle school	9,868,389	89.5	3.0 (2.1–4.4) **	90.3	2.5 (1.6–3.5) *
Economic development level of resident areas# (%)					
Low	2,989,523	80.4	1	82.0	1
Middle	3,560,671	87.2	2.2 (1.7–3.7) *	85.6	1.5 (0.8–3.2) *
High	4,361,336	92.5	3.7 (2.4–5.2) **	95.4	3.3 (2.5–4.5) **
Frequency of vaccination service (%)					
≤3 times per month	5,291,348	87.9	1	83.7	1
1–4 times per week	3,863,390	86.6	1.0 (0.8–1.0)	91.5	2.2 (1.5–3.9) *
≥5 times per week	1,756,792	87.9	1.0 (0.9–1.1)	96.4	3.4 (2.2–5.8) **
**Random effects**					
Variance (S.E.)	-	0.8 (0.13)		0.7 (0.12)	
ICC (%)	-	17.2		15.7	
**Model fit statistics**					
Log-likelihood	-	−216.5		−263.6	
AIC		240.2		263.5	

AOR: adjusted odds ratio. * *p* < 0.05. ** *p* < 0.01; ICC: intra class correlation; AIC: Akaike’s information criterion; S.E.: standard error.
